# Safety Pharmacology, Toxicokinetics and Pharmacodynamics of VMX-C001, an Intravenous Bypassing Agent for Factor Xa Inhibitors, in Animal Models

**DOI:** 10.3390/pharmaceutics18101210

**Published:** 2026-09-23

**Authors:** Daniël Verhoef, Gerard Short, Tainá Gomes, Kees Groen, Henri Spronk, Jan Ohrstrom, Pieter H. Reitsma

**Affiliations:** 1 VarmX, Emmy Noetherweg 2, Plus Ultra Building, 2333 BK Leiden, The Netherlandsp.h.reitsma@varmx.com (P.H.R.); 2 Division of Thrombosis and Haemostasis, Einthoven Laboratory for Vascular and Regenerative Medicine, Leiden University Medical Centre, Albinusdreef 2, 2333 ZA Leiden, The Netherlands; 3 DGr Pharma, Hoge Mosten 2c1, 4822 NH Breda, The Netherlands; 4 Department of Biochemistry, Cardiovascular Research Institute Maastricht, Maastricht University, Universiteitssingel 50, 6229 ER Maastricht, The Netherlands; 5 Thrombosis Expertise Center, Maastricht University Medical Center, P. Debyelaan 25, 6229 HX Maastricht, The Netherlands

**Keywords:** anticoagulation, VMX-C001, FXa inhibitor-bypassing agent, direct oral factor Xa (FXa) anticoagulant, preclinical study

## Abstract

**Background:** Anticoagulation therapy confers increased risk of potentially life-threatening bleeding events. Limited options exist for anticoagulation reversal in patients treated with direct oral factor Xa (FXa) anticoagulants (aka FXa inhibitors). Clinical development of reltodecacog alfa (VMX-C001), a modified form of human zymogen FX, as an FXa inhibitor-bypassing agent is ongoing. We report non-clinical pharmacology, toxicokinetic and pharmacodynamic studies of VMX-C001. **Methods:** Dose-ranging and 2-week toxicity studies were conducted in which Sprague–Dawley rats and cynomolgus monkeys received VMX-C001 at various doses for ≤2 weeks. Safety, pharmacologic, toxicokinetic and pharmacodynamic parameters were assessed. The anticoagulation-bypassing ability of VMX-C001 against edoxaban (ex vivo spiking) and rivaroxaban (in vivo) in monkeys was also evaluated. **Results:** No safety concerns were noted in either species at VMX-C001 doses up to 10 mg/kg; no VMX-C001-related adverse clinical signs were observed. VMX-C001 exhibited approximately dose-proportional kinetics, with no accumulation after repeat dosing. Changes in thrombin-generation parameters in both species were mild and non-adverse. VMX-C001 restored rivaroxaban-induced decreases in thrombin generation toward pre-rivaroxaban levels. In blood samples spiked ex vivo with edoxaban, the ability of VMX-C001 to support thrombin generation was maintained, even after repeat dosing. **Conclusions:** Treatment with VMX-C001 for 2 weeks was well tolerated at all tested doses. VMX-C001 had an approximately dose-proportional toxicokinetic profile, and accumulation was not observed upon repeat dosing. VMX-C001 also displayed a favourable pharmacodynamic profile and supported thrombin generation in the presence of FXa inhibitors.

## 1. Introduction

The coagulation cascade comprises a complex network of proteins that balance pro- and anticoagulant stimuli to protect the human body from thrombosis and bleeding. Blood coagulation factor X (FX) plays an important role in this system: in addition to other roles, activated FX (FXa) converts prothrombin to thrombin, which, in turn, helps to convert soluble fibrinogen to the insoluble fibrin strands that serve to stabilise the platelet-based primary blood clot [1,2,3].

Anticoagulation is advised for individuals with an increased risk of thromboembolic events; the historical standard of care was vitamin K antagonists (i.e., warfarin) or low-molecular-weight heparins [4,5]. In recent years, FXa inhibitors such as rivaroxaban, edoxaban and apixaban have become the standard of care to reduce the risk of thromboembolic complications in patients with nonvalvular atrial fibrillation and for the prevention and treatment of venous thromboembolism [4,5,6,7,8].

Anticoagulation therapy carries with it an increased risk of bleeding events, which can be life-threatening [9,10,11]. Patients at particular risk of bleeding include older people, particularly those who are frail [10,12,13]. In the case of non-life-threatening bleeding events occurring in patients receiving FXa inhibitors, adequate control may be achieved through supportive care, e.g., by temporarily withholding therapy [9]. However, prompt anticoagulation reversal is necessary in specific situations, e.g., when severe or life-threatening bleeding occurs or when an urgent or high bleeding-risk surgical procedure is needed [9,10,11].

Currently, the only specific anticoagulation reversal agent for FXa inhibitors is andexanet alfa, a recombinant modified human FXa protein indicated when reversal of anticoagulation is needed due to life-threatening or uncontrolled bleeding in patients being treated with rivaroxaban or apixaban [14,15]. Andexanet alfa competes with native FXa for binding of FXa inhibitors, thereby counteracting their anticoagulant activity [16]. However, this anticoagulation reversal agent was associated with thrombotic events in 10.3% of patients and has recently been withdrawn in the USA [17]. As such, there is an unmet need for a safe and effective reversal agent for FXa inhibitor-associated bleeding or for pre-surgery reversal of FXa inhibitor anticoagulation.

VMX-C001 is a recombinant, modified human coagulation FX that is in clinical development as an FXa inhibitor-bypassing agent [18,19]. VMX-C001 retains the core structure of endogenous FX, but a 7-amino-acid stretch in the serine protease domain has been replaced with a 16 amino-acid sequence derived from an FXa present in snake venom [20], rendering VMX-C001 insensitive to inhibition by FXa inhibitors. This allows it to function as an FXa inhibitor-bypassing agent by replacing inhibited natural FX in the coagulation process and restoring thrombin generation. It is therefore expected to enable safe and effective restoration of coagulation in the case of bleeding or emergency surgery in patients treated with FXa inhibitors [1].

Toxicity assessments in animals, conducted in accord with International Council for Harmonisation of Technical Requirements for Pharmaceuticals for Human Use (ICH) guidelines [21], are an integral part of drug development. Rats and non-human primates are widely used for this purpose, particularly for biological products [21,22]. ICH Guideline S6(R1) recommends that the following animal studies using a product comparable to that to be used in clinical studies be performed to inform clinical decision-making: dose-ranging studies to identify toxic doses and a no observed adverse effect level (NOAEL); pharmacokinetic and toxicokinetic studies; local tolerance studies; recovery studies to examine the reversibility of effects; and immunogenicity studies, e.g., of anti-drug antibodies (ADAs), to correlate antibody formation with pharmacokinetic/pharmacodynamic parameters and adverse effects [23]. Both single- and multiple-dose studies are recommended, as is the use of multiple assay methods where appropriate. The ICH does not specify the specific assays to be used but emphasises assay validation and reproducibility [21,23].

As part of the development of VMX-C001, we have assessed its safety, pharmacologic, toxicokinetic and pharmacodynamic profiles in Sprague–Dawley rats and cynomolgus monkeys (*Macaca fascicularis*).

## 2. Materials and Methods

### 2.1. Animals

Sprague–Dawley rats (Charles River Laboratories Italia, Calco, Italy) aged 11–14 weeks (dose range finding), 8–9 weeks (toxicity), or 8–11 weeks (respiratory function) at receipt were acclimatised to the study conditions for 7 (part 1 of dose range-finding study), 14 (part 2 of dose range finding), ≥14 (toxicity), or ≥11 (respiratory function) days before the beginning of the dosing period. They were allocated to groups using either a computerized randomization procedure (dose range finding, toxicity) or a computerized stratification procedure designed to ensure that the average body weight of each group was similar (respiratory function) either at receipt (toxicity) or during acclimatisation (dose range finding, respiratory function). The animals were housed in groups of up to three in polycarbonate cages containing autoclaved sawdust bedding. They were provided with rat huts and Nylabone for psychological and environmental enrichment, except when interrupted by study procedures. Food (rat/mouse pelleted maintenance diet; SSNIFF Spezialdiäten, Soest, Germany) and water were provided ad libitum, except during designated procedures.

Cynomolgus monkeys (Le Tamarinier, Tamarin, Mauritius) were selected for study because they were considered the most appropriate non-rodent species for the toxicology studies based on pharmacological effect. Monkeys > 24 weeks of age were quarantined for at least 4 weeks either at the breeder or the test facility, during and/or at the end of which each animal was given a complete clinical examination. All animals used in the study had documentation to confirm one negative tuberculosis test. The animals were acclimatised to the study conditions for ≥11 days (dose range finding) or ≥34 days (toxicity) before initiation of dosing, during which time pre-treatment investigations were performed. The animals were also trained to the restraining procedure (restraint chair) on two ≥15-min occasions during the pre-treatment period. Animals due to receive rivaroxaban were also administered drinking water by gavage three times during the pre-treatment period. Monkeys were housed in cages in groups and were provided with at least four items, such as toys, except when interrupted by study procedures. Food was provided once daily, at least 1 h after administration on days of dosing (Primate Leaf Eater vegetarian diet, SSNIFF Spezialdiäten GmbH). In addition, fruit was given daily, and animals were offered dried apricots as a reward following study-related procedures. Water was provided ad libitum. Humane endpoints were not used because all animals were expected to and did survive to the end of the studies.

Investigators were not blinded to the groups to which animals were assigned.

### 2.2. Materials

VMX-C001 (batch no. ENG-0007 [dose range finding] or CMC-O-0102 [toxicity and respiratory function]; VarmX, Leiden, The Netherlands) was a frozen liquid stored at a concentration of 10.4 mg/mL at −20 °C and protected from light. The vehicle for VMX-C001 (batch no. CMC-O-0091; pH 8.0) was a liquid comprising 10 mM arginine, 10 mM glutamic acid, 5% sorbitol and 0.01% polysorbate 80 and was stored at −20 °C. Before use, VMX-C001 and vehicle aliquots were thawed under aseptic conditions in a water bath at a maximum temperature of 37 °C, then kept on wet ice and manually re-homogenised before formulation.

Rivaroxaban (batch no. 607051927; Polpharma, Gdansk, Poland) was a light cream, crystalline powder that was stored at room temperature. Formulations were prepared on each day of administration, kept on wet ice and protected from light until use. Edoxaban tosylate (Daiichi Sankyo, Tokyo, Japan, batch no. 8073u-0016) was sourced from Alsachim (Illkirch-Graffenstaden, France).

### 2.3. Dose Range-Finding Studies

Escalating dose range-finding studies were conducted in Sprague–Dawley rats and cynomolgus monkeys to evaluate the potential toxicity of VMX-C001 and to guide the selection of dose levels for the 2-week toxicity studies. VMX-C001 doses were selected to reflect the expected dose to be used in human studies (2 mg/kg) and to allow higher doses (5 and 10 mg/kg) to be used to determine the NOAEL.

VMX-C001 was administered to rats by slow bolus intravenous (IV) injection to the tail vein over a 1 min period using a syringe fitted with a single-use plastic catheter (Venoflux 247.06; Vygon, Écouen, France). VMX-C001 was administered to cynomolgus monkeys by slow bolus IV injection to the saphenous vein over a 1 min period using a single-use plastic syringe (e.g., Terumo^®^ syringe without needle, Tokyo, Japan, 10 mL or 20 mL or similar) fitted with a sterile infusion set (e.g., Terumo^®^ winged infusion set, or similar). In part I, VMX-C001 was administered at successive doses of 2, 5 and 10 mg/kg/day on 2 consecutive days, followed by either a 5-day (rats, n = 8) or 3-day (monkeys, n = 4) wash-out period. In part II, VMX-C001 was administered using the same approach at a constant dose for either 8 (rats, n = 24) or 7 (monkeys, n = 4) days (Figure 1). Toxicity was assessed based on survival, clinical signs, body weight and food consumption. Animals were sacrificed at the end of the study period and examined post mortem (gross pathology, organ weights, and histopathology).

These studies were conducted in environments compliant with Good Laboratory Practice (GLP).

### 2.4. Two-Week Toxicity and Recovery Studies

Dose formulations for all injections were delivered at room temperature and were administered within 3 h. Formulations were gently homogenised by five manual inversions and were visually checked for homogeneity before administration. Doses were administered by slow bolus IV administration using the technique specified for the dose-ranging studies at approximately the same time of day on each dosing day.

Sprague–Dawley rats and cynomolgus monkeys were each assigned to one of four VMX-C001 dose groups: 0, 2, 5 or 10 mg/kg (Figure 1). In rats, VMX-C001 doses were administered daily for 2 weeks (n = 20 animals per group; male:female, 1:1), followed by a 2-week (no treatment) recovery period (0 and 10 mg/kg dose groups only; n = 10 animals per group; male:female, 1:1). In cynomolgus monkeys, VMX-C001 was administered once every third day for 2 weeks (main study; n = 6 animals per group; male:female, 1:1), followed by a 2-week (no treatment) recovery period (0 and 10 mg/kg dose groups only; n = 4 monkeys per group; male:female, 1:1). Only a proportion of animals treated at the highest VMX-C001 dose (10 mg/kg) entered the recovery period based on the absence of major safety findings in the dose-ranging studies. Study animals that did not enter the recovery period were euthanised on Day 15, while recovery animals were maintained untreated for 2 weeks, then euthanised (Day 29). Post-mortem examination of gross pathology, organ weights and histopathology was performed.

These studies were GLP-compliant.

### 2.5. Anticoagulation Studies

#### 2.5.1. Edoxaban Ex Vivo Anticoagulation Bypassing Study

The edoxaban anticoagulation bypassing study was performed ex vivo using citrated plasma samples spiked with an edoxaban concentration known to have maximum anticoagulant effect in thrombin generation. Samples from two male and two female cynomolgus monkeys who had received VMX-C001 2 mg/kg/day (the anticipated dose for use in human studies) repeatedly over a 10-day period (Days 1–2, 6–7 and 11–12) in part 1 of the dose range-finding study were used to assess coagulation in the presence of edoxaban (Figure 1). Plasma samples from Days 1, 6 and 11 were collected before and 5 min after VMX-C001 dosing. Thrombin generation was assessed in plasma samples spiked ex vivo with 250 ng/mL edoxaban using the same methods as described for thrombin generation (see below).

#### 2.5.2. Rivaroxaban in Cynomolgus Monkeys

For the anticoagulation-bypassing experiment, cynomolgus monkeys were used (n = 5) (Figure 1). Male monkeys were used to avoid any potential differences in rivaroxaban metabolism between males and females and to minimize the potential for factors such as weight to influence results. These monkeys received vehicle on Days 1, 4, 7 and 10 of the study and were anticoagulated on Day 13 using a single dose of rivaroxaban (10 mg/kg) administered by oral gavage (using a syringe with an attached plastic oesophageal tube). A single dose of VMX-C001 (5 mg/kg) was administered 4 h later. The rivaroxaban formulation was stirred continuously for at least 15 min before dosing, maintained under delivery conditions (room temperature), and administered within 4 h after the end of its preparation. Clinical chemistry parameters and thrombin generation were measured in these animals before rivaroxaban administration, just before administration of VMX-C001; 30 min after VMX-C001 (n = 5); and, finally, on Day 30 at the end of a recovery (no treatment) period (recovery study, n = 2). Animals that did not enter the recovery study were euthanised on Day 15; animals in the recovery study were euthanised on Day 30 (after sampling for clinical chemistry). Rivaroxaban plasma concentration was determined using an anti-Xa chromogenic method using the Biophen™ DiXal kit, reagents, and a Rivaroxaban Plasma Calibrator kit (Hyphen BioMed, Neuville Sur Oise, France).

### 2.6. Toxicokinetic Study

Toxicokinetic analyses were conducted using an additional group of rats (n = 60, male:female, 1:1) and on blood samples from monkeys in the toxicity and rivaroxaban anticoagulation studies (n = 29, male:female, 1:1).

### 2.7. Assessments

The schedule for toxicokinetic blood sample collection/parameter measurement in Sprague–Dawley rats and cynomolgus monkeys is shown in Supplementary Table S1. In general, blood samples were collected with a syringe previously saturated with a 3.2% sodium citrate solution and transferred to tubes containing 33.3 μL 3.2% sodium citrate solution. Tubes were gently inverted manually, then kept upright without shaking and placed on wet ice pending centrifugation. Further detail on sample processing by assessed parameter is provided in the Supplementary Materials.

### 2.8. Safety

The assessment of safety was based on survival, clinical signs, body weight, food consumption, electrocardiograms, respiratory function, clinical pathology (haematology, coagulation, clinical chemistry and urinalysis), histology and ADAs. A functional observational battery evaluation (detailed clinical examination and assessment of reactivity to manipulation and to different stimuli) was also conducted in rats. The effect of VMX-C001 (5 mg/kg) on cardiovascular parameters in cynomolgus monkeys was also evaluated, and a conscious rat study evaluated the effect of a single IV dose of VMX-C001 (2, 5 or 10 mg/kg) on respiratory parameters.

### 2.9. Immunogenicity

For the anti-VMX-C001 and anti-FX antibody assays, samples were diluted 10-fold in assay buffer without (screening) or with (confirmatory) an excess amount of unlabelled VMX-C001 or FX (2.5 μg/mL after dilution). A master-mix solution containing biotinylated VMX-C001 or FX and Sulfo-Tag-labelled VMX-C001 or FX (Ardena Bioanalysis, Assen, The Netherlands) was added to the diluted samples in a 2:1 ratio (*v*/*v*). After incubation, the samples were transferred to a blocked streptavidin-coated Meso Scale Discovery (MSD) plate (MSD, Baltimore, MD, USA), incubated and washed to remove unbound reagent. Read buffer was then added, and samples were analysed using a Meso^®^ QuickPlex SQ120 reader (MSD).

### 2.10. Toxicokinetics

The analysed toxicokinetic parameters included the area under the concentration curve over the observation period (AUC_last_), the concentration of VMX-C001 at the time of dosing (C_0_), total systemic drug clearance (C_L_), mean residence time (MRT), apparent elimination half-life (t_1/2_) and volume of distribution (V_d_). The non-compartmental toxicokinetic analysis was performed on the mean plasma concentrations per dose group using validated Phoenix WinNonlin version 8.1 software (Certara, Radnor, PA, USA; NCA model was applied).

### 2.11. D-Dimers and TAT in Cynomolgus Monkeys

D-dimers were detected by quantitative sandwich enzyme-linked immunosorbent assay using mouse monoclonal anti-D-dimer antibody coated on the internal walls of a plastic microplate well. Rabbit anti-human fragment D antibodies coupled with peroxidase were then bound to the remaining free antigenic determinants of the bound D-dimer. For the detection of thrombin-antithrombin (TAT), any TAT present in the sample binds to antibodies against thrombin attached to the surface of a plastic microplate. Unbound constituents were removed by washing; then, peroxidase-conjugated antibodies to human antithrombin (AT) were bound to free AT determinants. In both cases, the reaction was terminated by the addition of a strong acid. The resulting colour intensity was read at a wavelength of 450 nm for D-dimer and 492 nm for TAT using a Synergy™2 reader (BioTek Instruments, Winooski, VT, USA). Gen5™ Secure software (Agilent Technologies, Santa Clara, CA, USA) was used for data processing. Priority was given to analysis of D-dimer in the event of insufficient sample volume, which occurred in approximately 20% of samples due to technical difficulties in blood drawing.

Thrombin generation was measured in 3.2% citrated plasma using calibrated automated tomography (Thrombinoscope, Maastricht, The Netherlands). Thrombin generation was triggered with the addition of tissue factor, phospholipids and calcium ions (platelet-poor plasma (PPP) trigger reagent, Stago, Paris, France). A fluorogenic substrate was then added for real-time analysis of thrombin generation, and fluorescence was read using a Fluoroskan Ascent Fluorimeter (Thermo Fischer Scientific, Waltham, MA, USA). Plasma samples were calibrated against a known thrombin calibrator to correct for substrate depletion, sample colour and inner filter effect. Parameters that were analysed, in triplicate on each plate, included lag time, endogenous thrombin potential (ETP) and thrombin peak height.

### 2.12. No-Observed-Adverse-Effect Level

The VMX-C001 NOAEL dose was determined based on the study safety, immunogenicity and toxicokinetic findings.

### 2.13. Statistical Analyses

No statistical analysis was performed in the dose range-finding studies due to the absence of control groups.

In rats, descriptive statistics were used for means, standard deviations, percentages, the number of observations and/or incidences by dataset in the toxicity studies. Provantis version 9.3.0.0 software (Boston, MA, USA) was used for statistical analysis of body weight, food consumption, haematology, coagulation, clinical chemistry and urinalysis data. PATHDATA version 6.2 software (PDS Pathology Data Systems, Basel, Switzerland) was used for the statistical analysis of organ weight data. Parametric or non-parametric methodology was applied as appropriate.

For the studies in cynomolgus monkeys, the numbers of animals used, the duration of the studies, and analyses were based on ICH M3 [R2]—Guideline on Nonclinical Safety Studies for the Conduct of Human Clinical Trials and Marketing Authorization for Pharmaceuticals [24]. With the exception of analyses of cardiovascular function, descriptive statistics were used, and analyses were performed using Phoenix WinNonlin 8.1 (Certara) on parametric data. Data were considered to be normally distributed and variances as homogenous between groups. For each parameter, a two-way (time and treatment) analysis of variance (ANOVA) was performed for repeated measurements using SAS PROC MIXED version 9.2 via SAS Enterprise Guide version 4.2 (SAS Institute Inc., Cary, NC, USA). If a statistically significant treatment/time interaction was observed (*p* < 0.1), an additional one-way ANOVA was implemented at each time-point, followed by a Dunnett test (if one-way ANOVA *p* < 0.05). If the treatment/time interaction was not significant (*p* > 0.10) and a treatment effect was observed (*p* < 0.05), a Dunnett test was performed to compare the mean effect of each treatment (average of all time points). In cases where a significant difference was observed between pre-dose values, the described statistical comparisons were performed on individual variations from the baseline value of each animal and parameter.

## 3. Results

### 3.1. Dose Range-Finding Study

Repeated administration of VMX-C001 at ascending doses of up to 10 mg/kg once daily for 2 days (part 1) was well tolerated, inducing only minor non-adverse effects. Further study of 7 or 8 days of VMX-C001 dosing at a constant level of 10 mg/kg daily (part 2), chosen according to the part 1 findings, confirmed the tolerability results. Based on these results, doses of up to 10 mg/kg were used in the subsequent 2-week toxicity studies to assess the safety and pharmacologic, toxicokinetic and pharmacodynamic profiles of VMX-C001 in the same animal models.

### 3.2. General Safety

No safety concerns related to unscheduled deaths, clinical signs, body weight or food consumption, ophthalmic examination, haematology, coagulation, clinical chemistry or urinalysis assessments were noted in either species, either during dosing or the recovery periods. In rats, the reported clinical signs (e.g., swollen jugular vein, alopecia, and difficulty using forelimbs) were not considered to be related to VMX-C001. In monkeys, minor clinical signs included a moderate induration on the left saphenous vein, a nodule in the right axillary lymph node and a vitreous fuzzy area in the left eye. These clinical signs were not considered to be related to VMX-C001 because they were reported sporadically in single animals across dose groups; rather, these signs are considered to be due to repeated administration or blood sampling procedures and are common in group-housed animals.

In monkeys that received VMX-C001 and rivaroxaban, there was a mild transient decrease in urea concentration and increases in phosphorous, triglycerides and glucose. These changes were not seen when VMX-C001 was administered alone and were therefore considered to be due to rivaroxaban (either alone or in combination with VMX-C001).

In rats, no VMX-C001–related macroscopic findings, microscopic findings or changes in organ weight were found at necroscopy at the end of the treatment or recovery periods. Similarly, macroscopic necropsy findings in monkeys were judged to be related to the injection procedure, and no VMX-C001–related changes in organ weight were noted. Microscopic examination in monkeys showed acute inflammation characterized by minimal to moderate infiltrates of mixed inflammatory cells (neutrophils mainly) in perivenous/subcutaneous locations at the injection sites in all monkeys treated with VMX-C001 and in monkeys treated with VMX-C001 and rivaroxaban. These changes were reversible and considered indicative of a mild local irritant effect. Haemorrhage at the injection site was more pronounced in monkeys treated with VMX-C001 and rivaroxaban compared with monkeys treated with control and VMX-C001 alone, but reversible. In view of the anticoagulant effect of rivaroxaban, a relationship could not be excluded. Other microscopic findings were considered incidental because they also occurred in controls, were isolated or of low incidence, had no dose relationship in incidence or severity, and/or are common background findings for the monkey. The effects were judged to be non-adverse.

### 3.3. Respiratory Safety

The conscious rat study found that a single IV dose of VMX-C001 (2, 5 or 10 mg/kg) did not cause any change in respiratory parameters, including respiratory rate, peak inspiratory and expiratory flows, tidal and minute volumes, and enhanced pause.

### 3.4. Cardiovascular Safety

VMX-C001 5 mg/kg did not cause any change in cardiovascular parameters, including heart rate and electrocardiograph parameters, in cynomolgus monkeys.

### 3.5. Immunogenicity

No ADAs were observed pre-treatment or on Day 15 in rats or pre-treatment in monkeys. The animals with confirmed positive ADA results during the two studies are shown in Table 1. One female rat previously treated with VMX-C001 10 mg/kg tested positive for anti-FX antibodies, and another tested positive for both anti-FX and anti-VMX-C001 antibodies, both on Day 29. One female monkey treated with VMX-C001 10 mg/kg tested positive for anti-VMX-C001 antibodies, and another tested positive for both anti-VMX-C001 and anti-FX antibodies, both on Day 15. On Day 29, one male monkey in the VMX-C001 10 mg/kg group tested positive for anti-VMX-C001 antibodies, and one female tested positive for both anti-VMX-C001 and anti-FX antibodies.

### 3.6. Toxicokinetics

The toxicokinetic parameters of VMX-C001 in male and female rats and monkeys after single or repeated dosing over 14 days are presented in Table 2. There were no substantial differences between males and females in either species, and no accumulation was observed after repeated dosing for 2 weeks. In rats, VMX-C001 showed approximately dose-proportional kinetics over the dose range of 2–10 mg/kg based on accumulation ratios, with males showing slightly higher exposure than females, particularly after 14 days of dosing. A 5-fold increase in dose from 2 mg/kg to 10 mg/kg resulted in a 4.6- to 6.1-fold increase in VMX-C001 exposure, with a 2.2- to 2.6-fold increase between 2 mg/kg and 5 mg/kg and a 2.0- to 2.4-fold increase between 5 mg/kg and 10 mg/kg; exposure ratios based on AUC_last_ in male versus female rats ranged from 1.1 to 1.5. The accumulation ratios based on AUC_last_ ranged between 1.1 and 1.3 for Day 14 vs. Day 1.

In monkeys, a 5-fold increase in dose from 2 mg/kg to 10 mg/kg resulted in a 5.8- to 6.5-fold increase in VMX-C001 exposure, with a 2.1- to 2.9-fold increase between 2 mg/kg and 5 mg/kg and a 2.2- to 2.7-fold increase between 5 mg/kg and 10 mg/kg. Exposure ratios based on AUC_last_ in male versus female rats ranged from 0.9 to 1.2. Accumulation ratios based on AUC_last_ ranged between 0.8 and 1.0 for Day 13 vs. Day 1. Simultaneous administration of VMX-C001 with rivaroxaban on Day 13 did not influence the toxicokinetics of VMX-C001 dosed 4 h after rivaroxaban administration.

Data for monkeys that screened positive for antibodies against FX and/or VMX-C001 on Day 15 were compared with Day 13 data for these animals. This showed limited exposure of VMX-C001 on Day 13, an effect that was also observed in the individual plasma concentration–time curves (Supplementary Table S2).

### 3.7. D-Dimers and TAT in Cynomolgus Monkeys

D-dimer reflects fibrin formation and subsequent degradation, and TAT reflects thrombin generation/inactivation; these are useful markers of activated coagulation and clot resolution. As such, these markers were assessed to screen for potential prothrombotic side effects of VMX-C001. No relevant VMX-C001-related changes in D-dimer or TAT concentrations were observed at any dose level (Figure 2). In monkeys receiving VMX-C001 only, mild increases in D-dimers were observed in a few individuals; these increases were of a similar magnitude between groups but occurred at a higher incidence at 10 mg/kg.

### 3.8. Thrombin Generation

#### 3.8.1. VMX-C001 in Rats and Monkeys

Administration of VMX-C001 in rats was associated with non-significant changes in thrombin-generation parameters at doses ≥ 2 mg/kg (Supplementary Figure S1A). Similarly, administration of VMX-C001 to monkeys was also associated with only non-significant changes in thrombin-generation parameters (Supplementary Figure S1B), including peak height and lag time, which were increased in individual monkeys at various doses and time points.

#### 3.8.2. VMX-C001 and Edoxaban in Cynomolgus Monkeys (Ex Vivo)

In plasma samples taken from monkeys on Days 1, 6 and 11 and spiked ex vivo with edoxaban, VMX-C001 maintained the thrombin generation lag time and peak height at near-baseline levels, regardless of repeat dosing (Figure 3).

#### 3.8.3. VMX-C001 and Rivaroxaban in Cynomolgus Monkeys (In Vivo)

Treatment of cynomolgus monkeys with 10 mg/kg rivaroxaban by oral gavage was associated with markedly suppressed thrombin generation parameters. However, administration of 5 mg/kg VMX-C001 restored thrombin generation toward pre-rivaroxaban levels (Figure 4). Mean plasma concentrations of rivaroxaban at 0 and 30 min were 378 ng/mL and 437 ng/mL, respectively, indicating that its inhibitory activity was maintained over this time period.

No relevant treatment-related changes in D-dimer concentrations were noted compared with respective pre-treatment values and control animals (Supplementary Figure S2). However, one monkey did have a moderate increase in TAT 30 min and 4 h after VMX-C001 administration on Day 13 and on Day 15 (Supplementary Figure S2). These changes were considered to be related to VMX-C001 administration following rivaroxaban, as they correlated with the recovery of rivaroxaban-induced thrombin generation changes. No other changes were considered related to VMX-C001, based on their small magnitude (or a magnitude of change commonly observed in monkeys under similar study conditions), inconsistent direction, absence of a dose response and general overlap of individual values with the range of control and baseline values.

### 3.9. No-Observed-Adverse-Effect Level

Under the experimental conditions of this study, VMX-C001 induced only non-adverse minor changes of clinical pathology parameters and non-adverse local effects at the injection site at any of the doses tested in both rats and monkeys, with no differences between males and females. Therefore, the NOAEL for VMX-C001 was determined to be 10 mg/kg, the highest dose tested, in both sexes and animal species. At the NOAEL, the mean C_0_ and last-observed AUC values (not dose-normalised) in rats were 224.4 μg/mL and 759.4 μg·h/mL for males and 172.3 μg/mL and 659.7 μg·h/mL for females, respectively, after 2 weeks of treatment. For cynomolgus monkeys (male and female), mean last-observed AUC at the NOAEL was 1411 μg·h/mL.

## 4. Discussion

This programme of non-clinical studies was designed to characterise the safety pharmacology, toxicokinetics and pharmacodynamics of VMX-C001, a modified recombinant human factor X zymogen being developed as a bypassing agent for FXa inhibitors, independent of inhibitor type. Collectively, the data demonstrate that repeat IV administration of VMX-C001 at doses up to 10 mg/kg for up to 2 weeks was well tolerated in both Sprague–Dawley rats and cynomolgus monkeys, with no adverse clinical or pathological findings and no consistent biomarker evidence of systemic coagulation activation under the studied experimental conditions. Importantly, VMX-C001 preserved or restored thrombin-generation capacity in the presence of FXa inhibitors edoxaban and rivaroxaban, both ex vivo and in vivo, supporting its proposed mechanism of action as a functional replacement for inhibited endogenous factor X [1,18,25].

VMX-C001 did not induce treatment-related mortality; clinically relevant changes in body weight, food intake or clinical pathology parameters; or adverse macroscopic or microscopic findings in either species in any of the performed studies. Observed inflammatory changes at injection sites in monkeys were mild, fully reversible and consistent with local administration-related effects commonly observed following repeated IV dosing of biological products [26], rather than a systemic toxicological signal. No VMX-C001-related adverse respiratory or cardiovascular effects were detected in dedicated safety pharmacology studies, further supporting a favourable non-clinical safety profile in line with ICH S6(R1) guidance for biotechnology-derived therapeutics [21,23].

Of particular relevance to haemostatic safety, VMX-C001 did not induce sustained or dose-dependent increases in circulating D-dimers or TAT complexes. These biomarkers are sensitive markers of in vivo thrombin generation and fibrin turnover [27,28], and the absence of consistent elevation suggests that VMX-C001 does not promote uncontrolled coagulation activation under the studied experimental conditions. The isolated, transient TAT increase observed in a single monkey following rivaroxaban and VMX-C001 co-administration coincided with restoration of thrombin generation and was not associated with downstream coagulopathy or pathological sequelae. Taken together, these findings indicate that no consistent coagulation-activation signal was detected, a key theoretical risk for bypassing agents [29].

VMX-C001 displayed apparently dose-proportional toxicokinetics in both species across the tested dose range based on accumulation ratios, with no evidence of accumulation following repeat dosing. Systemic exposure increased approximately proportionally with dose, and variability between sexes was limited. Elimination half-life and mean residence time were consistent with those reported for other coagulation factors administered intravenously and support intermittent dosing suitable for acute or peri-procedural use [16]. The absence of accumulation is particularly relevant for the envisioned clinical use of VMX-C001 in emergency settings, where single or short-course administration is anticipated. Moreover, co-administration of rivaroxaban did not alter the toxicokinetic profile of VMX-C001, suggesting a low likelihood of pharmacokinetic drug–drug interactions with FXa inhibitors. This independence contrasts mechanistically with sequestration-based reversal strategies [10] and may simplify clinical use in patients with variable or unknown anticoagulant exposure.

Anti-factor X antibodies were detected in animals at all VMX-C001 doses after repeat dosing, whereas anti-VMX-C001 antibodies were detected primarily at the highest VMX-C001 dose tested. VMX-C001 has 98% sequence identity with human FX. Therefore, anti-FX antibody-positive samples are expected to cross-react with both anti-FX and VMX-C001. The high-titre anti-VMX-C001 antibodies are likely due to the snake-derived insertion in the serine protease domain, which is unique to VMX-C001. Overall, based on the intended use of VMX-C001 as a single-administration drug, ADAs are of limited concern.

While ADA-positive animals exhibited reduced plasma VMX-C001 exposure, no functional consequences for coagulation parameters or safety endpoints were observed. This pattern is consistent with immune responses occasionally seen with human proteins administered to non-human species and does not preclude clinical development, particularly given the short-term, situational use anticipated for VMX-C001 [23,30]. Notably, Phase 1 clinical data indicate low immunogenicity in humans following single-dose exposure [18], supporting the translational relevance of the non-clinical findings.

VMX-C001 induced mild, non-adverse changes in endogenous thrombin-generation parameters when administered alone. In both rats and monkeys, the overall capacity to generate thrombin (endogenous thrombin potential and peak height) was preserved, although modest prolongation of lag time was observed at higher doses. These changes likely reflect physiological rebalancing of pro- and anticoagulant forces following introduction of an FX variant rather than a deleterious pharmacodynamic effect. Importantly, these alterations did not translate into biomarker evidence of pathological coagulation activation.

The most clinically informative findings emerged from the FXa inhibitor-bypassing experiments. Ex vivo edoxaban spiking experiments demonstrated that VMX-C001 consistently supported thrombin generation across multiple dosing days, indicating sustained bypass capacity, even after repeat exposure. In cynomolgus monkeys, rivaroxaban produced the expected marked suppression of thrombin generation; VMX-C001 administration restored thrombin generation toward pre-rivaroxaban levels within 30 min of administration without concomitant increases in D-dimers, indicating an absence of a drug–drug interaction on prothrombotic markers. Rivaroxaban concentrations were maintained between 0 and 30 min after dosing in the presence of VMX-C001. Importantly, these data align with mechanistic studies demonstrating that VMX-C001 retains procoagulant activity while being resistant to FXa inhibitors [1,31,32].

Unlike andexanet alfa, which neutralises FXa inhibitors through direct binding and requires continuous infusion to maintain effect [14,15], VMX-C001 functions downstream by replacing inhibited FX within the coagulation cascade [1]. By restoring thrombin generation without depleting or sequestering anticoagulants, VMX-C001 offers a more physiologically integrated approach to haemostatic restoration. Furthermore, the absence of sustained increases in thrombin generation markers in these studies contrasts with the prothrombotic signal observed with andexanet alfa in clinical use [17,33], although direct comparative conclusions cannot be drawn from non-clinical data alone.

Despite advances in anticoagulation management, safe, rapidly acting and broadly effective strategies to address FXa inhibitor-associated bleeding and urgent surgical scenarios are still needed [9,10]. Andexanet alfa is currently approved only for apixaban- and rivaroxaban-associated bleeding [14,15,16,17,33], and ciraparantag, a small-molecule universal reversal agent that operates through non-specific electrostatic interactions, remains investigational [34,35,36,37,38]. The non-clinical data for VMX-C001 presented here support its role as a bypassing agent rather than a reversal or sequestration agent and suggest that it may restore thrombin generation without inducing systemic coagulation activation based on the lack of consistent biomarker evidence under the studied experimental conditions. Importantly, bypassing may be useful in situations where FXa inhibitors such as edoxaban are used and cannot be managed using currently approved agents. Furthermore, VMX-C001 may have utility in clinical contexts where rapid yet transient restoration of haemostasis is required; this is indicated by the rapid onset of restoration of thrombin generation parameters and the half-life of approximately 5 h in animals. The ability of VMX-C001 to function independently of the specific FXa inhibitor may also simplify clinical emergency decision-making when the drug identity or exposure level is unknown.

The studies reported here provide a non-clinical foundation that supports continued clinical development of VMX-C001. The absence of dose-limiting toxicities, favourable toxicokinetics, lack of accumulation and consistent pharmacodynamic bypass activity in animals informed the design and initiation of first-in-human studies. Indeed, a subsequently performed Phase 1 clinical study has demonstrated that VMX-C001 is well tolerated in healthy volunteers, exhibits predictable pharmacokinetics and restores thrombin generation in the presence of apixaban and rivaroxaban, consistent with the animal findings [18]. An ongoing phase 3 clinical study will determine the safety and efficacy of VMX-C001 in patients with active bleeding or undergoing urgent procedures who are taking FXa inhibitors [19]. The validation of sensitive coagulation assays that have been developed to monitor FXa inhibitor activity and bypassing effects will be critical to these programmes [31].

The strengths of these non-clinical studies, predominantly performed under GLP principles, include evaluation in two relevant species, incorporation of repeat-dose and recovery phases, and integration of mechanistic pharmacodynamic biomarkers with standard toxicology endpoints. While the present studies do not address long-term or chronic exposure, such use is not envisaged for VMX-C001 and is therefore not a limitation relative to its intended clinical role. Limitations include the inherent species differences in coagulation biology and immunogenicity and the controlled experimental context relative to the complex clinical scenarios in which FXa inhibitor reversal is required. Nevertheless, these limitations are common to the development of coagulation biologics and do not detract from the translational relevance of the findings.

## 5. Conclusions

In conclusion, VMX-C001 demonstrated a favourable safety, toxicokinetic and pharmacodynamic profile following repeat IV administration in rats and cynomolgus monkeys. The ability of VMX-C001 to restore thrombin generation in the presence of FXa inhibitors supports its continued clinical development as an anticoagulant-bypassing agent. These findings provide mechanistic and translational justification for the advancement of VMX-C001 into later-phase clinical studies of its use to manage FXa inhibitor-associated bleeding [19].

## Figures and Tables

**Figure 1:**
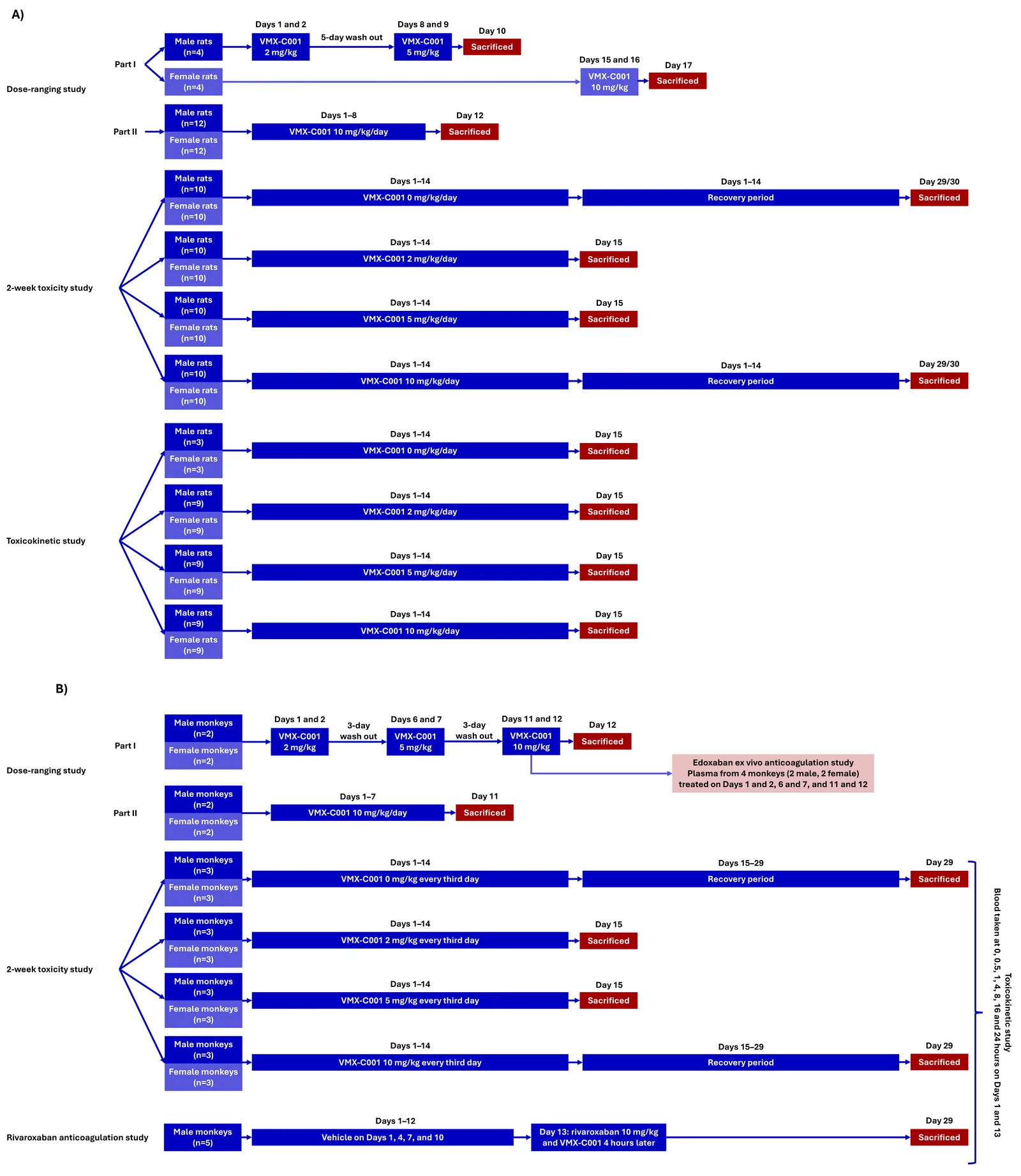
Dose-ranging and toxicokinetic studies in (**A**) rats and (**B**) monkeys.

**Figure 2:**
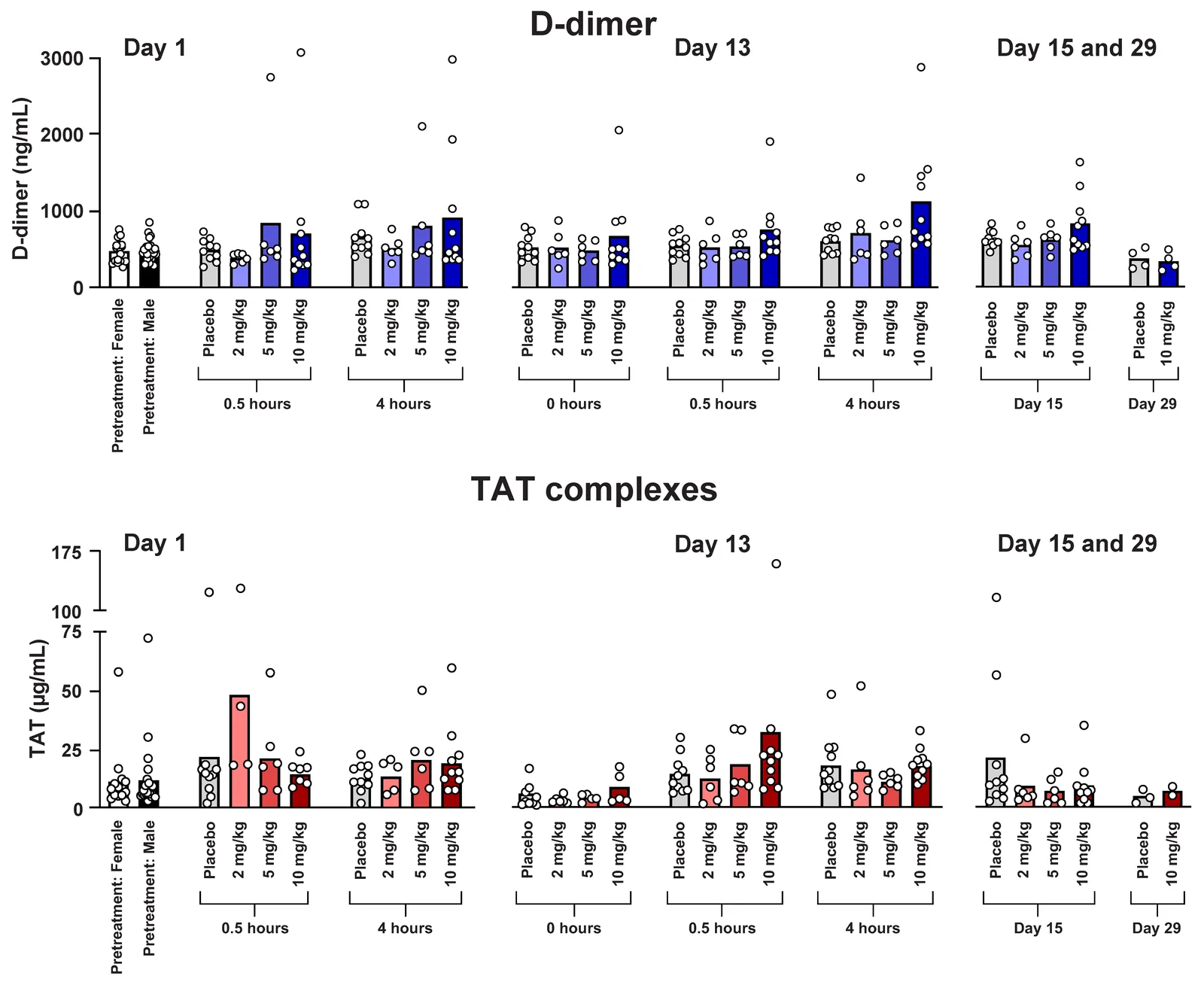
D-dimer and TAT complexes in cynomolgus monkeys treated with VMX-C001 (up to n = 6 per dose; male:female, 1:1 up to day 13; n = 4 per dose; male:female 1:1 up to days 13, 15 and 29). The bars show mean values, with individual values shown as open circles. Data from female and male monkeys are shown together for all time points except for pretreatment.

**Figure 3:**
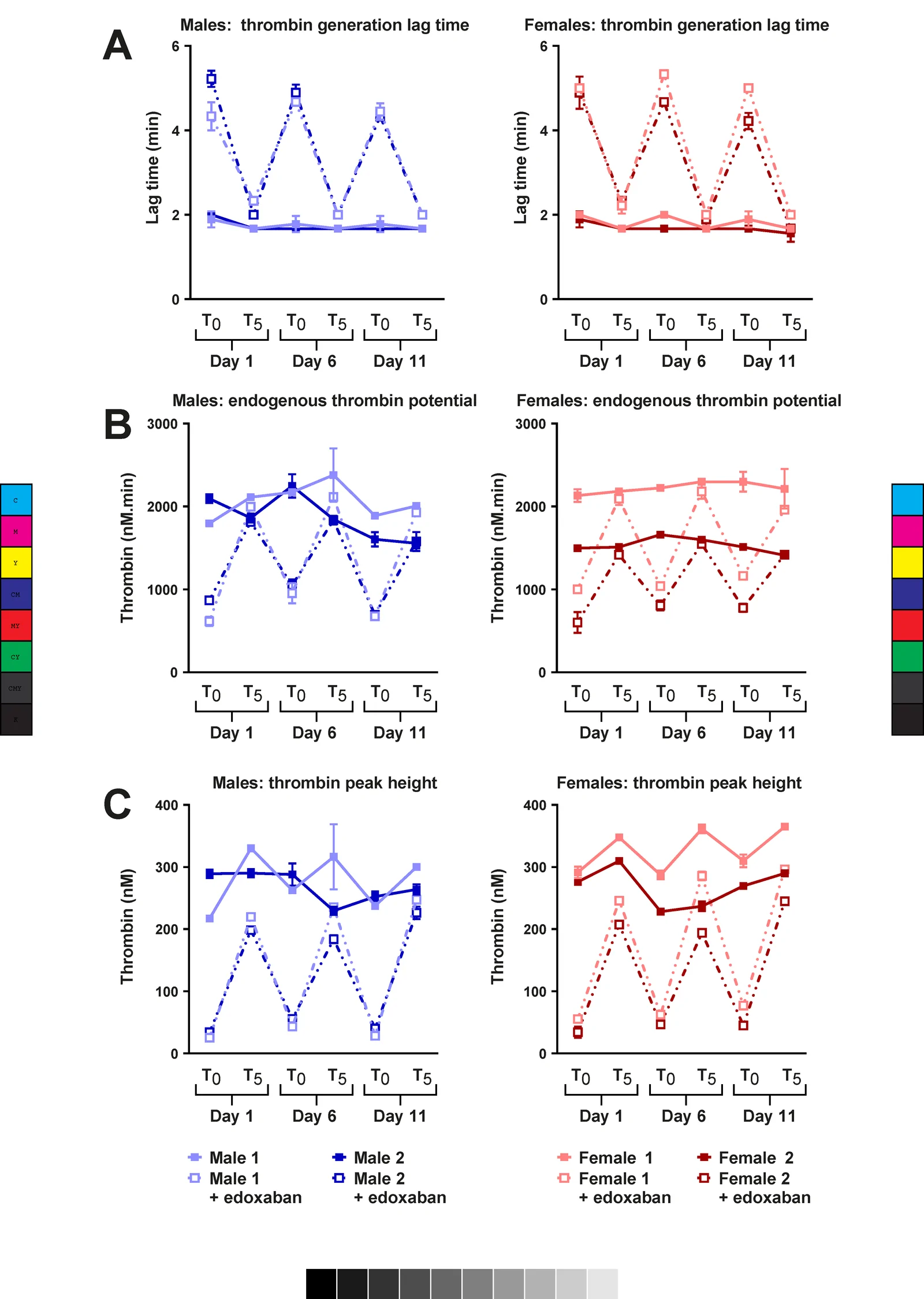
VMX-C001 (2 mg/kg IV) and edoxaban (dose; spiking) effects on thrombin generation in plasma from cynomolgus monkeys (n = 4). (**A**) Mean lag time in 2 male (left panel) and 2 female (right panel) monkeys (different coloured lines represent individual monkeys) before VMX-C001 administration (T_0_) and 5 min after VMX-C001 administration (T_5_) on Days 1, 6 and 11. The dotted lines represent values in the presence of edoxaban. As above for (**B**) mean endogenous thrombin potential and (**C**) mean thrombin peak height. Data points show the mean of three technical replicate measurements per sample. T_0_, before treatment; T_5_, 5 min after dosing.

**Figure 4:**
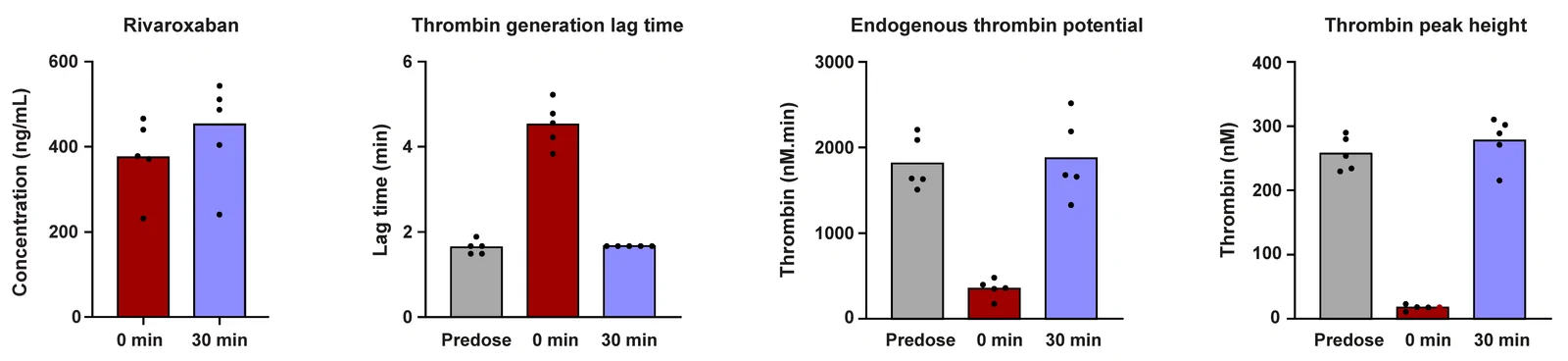
Mean thrombin generation parameters upon bypassing of rivaroxaban with VMX-C001 in cynomolgus monkeys (n = 5). The bars show mean values, with individual values shown as closed circles.

**Table 1:** Anti-drug antibodies in Sprague–Dawley rats and cynomolgus monkeys treated with VMX-C001.

Sex	Study Group	Study Day	Titre	
			Anti-VMX-C001 Antibodies	Anti-Factor X Antibodies
**Rats**				
Female	10 mg/kg	End of treatment- free period (day 29)	8	8
Female	10 mg/kg	End of treatment- free period (day 29)	1	–
**Cynomolgus monkeys**				
Female	Control	15 29		2 1
Female	2 mg/kg	15	–	16
Male	2 mg/kg	15	–	32
Male	2 mg/kg	15	–	4
Female	5 mg/kg	15	–	32
Male	5 mg/kg	15	–	16
Male	5 mg/kg	15	–	8
Male	10 mg/kg	15 29	–	8 8
Female	10 mg/kg	15 29	– 512	16 32
Female	10 mg/kg	15	8	–
Female	10 mg/kg	15 29	128 * 64	16 16
Male	10 mg/kg	29	128 *	–
Male	10 mg/kg (after rivaroxaban)	30	64	16

* Best estimate.

**Table 2:** Toxicokinetic parameters of VMX-C001 in Sprague–Dawley rats and cynomolgus monkeys after the first and last dose.

	Day 1						Day 13 (Monkeys)/14 (Rats)					
Dose	C_0_ (µg/mL)	AUC_last,DN_ (µg·h/mL)	MRT (h)	t_1/2_ (h)	CL (mL/h/kg)	V_d_ (mL/kg)	C_0_ (µg/mL)	AUC_last,DN_ (µg·h/mL)	MRT (h)	t_1/2_ (h)	CL (mL/h/kg)	V_d_ (mL/kg)
Male Sprague–Dawley rats (N = 10 per dose group)												
2 mg/kg	34.4	64.0	4.4	4.7	15.3	104.8	40.2	80.6	5.8	5.6	12.4	100.4
5 mg/kg	85.7	58.6	4.3	4.4	16.8	107.6	90.7	69.6	5.5	4.8	14.4	98.8
10 mg/kg	175.0	58.9	4.3	5.0	16.7	119.7	224.4	75.9	5.2	5.0	13.2	94.8
Female Sprague–Dawley rats (N = 10 per dose group)												
2 mg/kg	33.6	50.0	4.1	5.3	19.6	150.5	18.628.5	53.8	4.9	4.6	18.6	124.7
5 mg/kg	94.6	50.0	4.0	5.0	19.7	140.9	8118.0.7	55.5	5.3	5.3	18.0	138.0
10 mg/kg	192.5	53.9	4.2	5.0	18.2	132.8	172.315.2	66.0	5.3	4.6	15.2	101.5
Male cynomolgus monkeys (N = 3 per dose group)												
2 mg/kg	44.6	138.3	6.9	6.2	7.0	62.9	43.8	106.4	3.2	2.3	10.6	55.4
5 mg/kg	94.5	140.4	7.3	6.9	6.5	64.3	84.7	123.7	7.8	5.2	8.2	60.8
10 mg/kg	190.0	165.5	7.5	7.0	5.5	55.9	231.0	138.9	9.2	6.5	8.4	67.2
Female cynomolgus monkeys (N = 3 per dose group)												
2 mg/kg	36.2	127.6	7.4	7.8	7.4	83.1	40.1	123.6	7.5	5.4	8.2	71.4
5 mg/kg	88.7	134.8	7.3	7.4	6.7	71.5	102.9	105.0	6.85	5.0	12.1	78.2
10 mg/kg	200.5	166.8	7.4	7.0	5.6	56.2	236.7	143.3	7.5	5.0	7.1	49.2

AUC_last,DN_, area under the concentration–time curve from the time of dosing to the last observed concentration, dose-normalised; C_0_, concentration at time of dosing; CL, total systemic drug clearance; MRT, mean residence time; t_1/2_, apparent elimination half-life; V_d_, volume of distribution.

## Data Availability

Data supporting the findings of this study are available from the corresponding author upon reasonable request.

## Supplementary Materials

The following supporting information can be downloaded at: https://www.mdpi.com/article/10.3390/pharmaceutics18101210/s1. Table S1. Sample collection in Sprague–Dawley rats and cynomolgus monkeys by assessment; Table S2. Calculated toxicokinetic parameters. Supplementary Methods: Descriptions of sample processing for detection of anti-drug antibodies, toxicokinetics, TAT and D-dimers, thrombin generation, and rivaroxaban studies; Figure S1. Thrombin generation in Sprague–Dawley rats and cynomolgus monkeys treated with VMX-C001. Figure S2. D-dimer and TAT complexes upon bypassing of rivaroxaban with VMX-C001 in cynomolgus monkeys.
