# Supplementary material for: Safety Pharmacology, Toxicokinetics and Pharmacodynamics of VMX-C001, an Intravenous Bypassing Agent for Factor Xa Inhibitors, in Animal Models
Source: Pharmaceutics. 2026 Sep 23;18(10):1210. doi: 10.3390/pharmaceutics18101210 (PMC13708372; doi:10.3390/pharmaceutics18101210)

Supplementary Materials: Safety pharmacology, toxicokinetics and pharmacodynamics of VMX C001, an intravenous bypassing agent for factor Xa inhibitors, in animal models

Supplementary Table S1 Sample collection in Sprague-Dawley rats and cynomolgus monkeys by assessment

Study day	Time relative to administration*	Parameters
Sprague-Dawley rats		
Pre-treatment	-	Anti-drug antibodies
Day 1	Before	Thrombin generation<br>Toxicokinetics
	5 min after	Thrombin generation<br>Toxicokinetics
	30 min after	Toxicokinetics
	1 h after	Toxicokinetics
	4 h after	Toxicokinetics
	9 h after	Toxicokinetics
	16 h after	Toxicokinetics
	24 h after	Toxicokinetics
Day 14	Before	Thrombin generation<br>Toxicokinetics
	5 min after	Thrombin generation<br>Toxicokinetics
	30 min after	Toxicokinetics
	1 h after	Toxicokinetics
	4 h after	Toxicokinetics
	9 h after	Toxicokinetics
	16 h after	Toxicokinetics
	24 h after	Toxicokinetics
	Before necropsy	Toxicokinetics
Day 15	Before necropsy (jugular vein)	Anti-drug antibodies
	At necropsy (abdominal aorta)	Coagulation<br>Clinical chemistry<br>Urinalysis
Day 29/30 (recovery animals only)	Before necropsy (jugular vein)	Anti-drug antibodies
	At necropsy (abdominal aorta)	Coagulation<br>Clinical chemistry<br>Toxicokinetics

Study day	Time relative to administration*	Parameters
		Urinalysis
Cynomolgus monkeys		
Pre-treatment	–	Haematology<br>Coagulation<br>Clinical chemistry<br>Urinalysis<br>Thrombin-anti-thrombin<br>D-dimer<br>Anti-drug antibodies
Day 1	Before	Thrombin generation<br>Toxicokinetics
	5 min after	Thrombin generation<br>Toxicokinetics
	30 min after	Thrombin-anti-thrombin<br>D-dimer<br>Toxicokinetics
	1 h after	Toxicokinetics
	4 h after	Thrombin-anti-thrombin<br>D-dimer<br>Toxicokinetics
	9 h after	Toxicokinetics
Day 2	16 h after	Toxicokinetics
	24 h after	Toxicokinetics
Day 13	Before	Thrombin generation<br>Thrombin-anti-thrombin<br>D-dimer<br>Toxicokinetics
	5 min after	Thrombin generation<br>Toxicokinetics
	30 min after	Thrombin-anti-thrombin<br>D-dimer<br>Toxicokinetics
	1 h after	Toxicokinetics
	4 h after	Thrombin-anti-thrombin<br>D-dimer<br>Toxicokinetics
	9 h after	Toxicokinetics
Day 14	16 h after	Toxicokinetics
	24 h after	Toxicokinetics
Day 15	–	Haematology<br>Coagulation<br>Clinical chemistry<br>Urinalysis

Study day	Time relative to administration*	Parameters
		Thrombin-anti-thrombin<br>D-dimer<br>Anti-drug antibodies<br>Toxicokinetics<br>Blank matrix
Day 29 (recovery animals only)	Before necropsy	Haematology<br>Coagulation<br>Clinical chemistry<br>Urinalysis<br>Thrombin-anti-thrombin<br>D-dimer<br>Anti-drug antibodies<br>Toxicokinetics<br>Blank matrix

*Note that blood from control animals for toxicokinetic studies was taken only at 5 minutes post-dose

Supplementary Table S2 Calculated toxicokinetic parameters

Parameter	C_0 (µg/mL)	$C_{0, DN}$ (µg/mL)	AUC_{last} (µg.h/mL)	$AUC_{last, DN}$ (µg.h/mL)	AUC_{∞} (µg.h/mL)	MRT (h)	$t_{1/2}$ (h)	CL (mL/h/kg)	V_d (mL/kg)
**Day 1 - Males**									
2 mg/kg (all animals)	44.56	22.28	276.6	138.3	284.4[$]	6.949	6.197[$]	7.033[$]	62.88[$]
5 mg/kg (all animals)	94.51	18.90	701.9	140.4	773.0	7.302	6.893	6.498	64.34
10 mg/kg (all animals)	190.0	19.00	1656	165.6	1825	7.501	7.024	5.521	55.88
**Day 1 - Females**									
2 mg/kg (all animals)	36.22	18.11	255.2	127.6	272.3	7.355	7.845	7.399	83.05
5 mg/kg (all animals)	88.69	17.74	673.9	134.8	748.5	7.329	7.413	6.687	71.54
10 mg/kg (all animals)	200.5	20.05	1668	166.8	1849	7.427	7.000	5.616	56.23
**Day 13 - Males**									
2 mg/kg (all animals)	43.78	21.89	212.8	106.4	-	3.207	2.322	10.59	55.37
anti-VMX-C001 Abs*	43.78	21.89	212.8	106.4	-	3.207	2.322	10.59	55.37
anti-FactorX Abs**	38.04[$]	19.02[$]	308.1[$]	154.0[$]	-	-	-	6.492[$]	79.45[$]
5 mg/kg (all animals)	84.73	16.95	618.7	123.7	-	7.767	5.235	8.243	60.75
anti-VMX-C001 Abs*	84.73	16.95	618.7	123.7	-	7.767	5.235	8.243	60.75
anti-FactorX Abs**	97.30[$]	19.46[$]	738.5[$]	147.7[$]	-	10.03[$]	6.987[$]	6.771[$]	68.25[$]
10 mg/kg (all animals)	231.0	23.10	1389	138.9	-	9.220	6.546	8.422	67.21
anti-VMX-C001 Abs*	231.0	23.10	1389	138.9	-	9.220	6.546	8.422	67.21
anti-FactorX Abs**	237.6	23.76	1583	158.3	-	10.72	7.598	6.454	70.28
5 mg/kg, rivaroxaban (all an.)	109.1	21.82	721.0	144.2	-	8.897	6.435	6.975	70.22
anti-VMX-C001 Abs*	109.1	21.82	721.0	144.2	-	8.897	6.435	6.975	70.22
anti-FactorX Abs**	109.1	21.82	721.0	144.2	-	8.897	6.435	6.975	70.22
**Day 13 - Females**									
2 mg/kg (all animals)	40.12	20.06	247.2	123.6	-	7.546	5.353	8.233	71.37
anti-VMX-C001 Abs*	40.12	20.06	247.2	123.6	-	7.546	5.353	8.233	71.37
anti-FactorX Abs**	41.17	20.59	268.6	134.3	-	6.402[$]	9.079[$]	7.454	76.65
5 mg/kg (all animals)	102.9	20.59	525.1	105.0	-	6.808	5.014	12.14	78.17
anti-VMX-C001 Abs*	102.9	20.59	525.1	105.0	-	6.808	5.014	12.14	78.17
anti-FactorX Abs**	99.49	19.90	671.8	134.4	-	11.48[$]	8.308[$]	7.447	90.56
10 mg/kg (all animals)	236.7	23.67	1433	143.3	-	7.515	4.964	7.074	49.24
anti-VMX-C001 Abs*	191.5	19.15	1445	144.5	-	8.707	5.764	7.074	56.90
anti-FactorX Abs**	191.8	19.18	1531	153.1	-	9.546	6.439	6.588	61.09

[$]: Mean = individual parameter result

*: mean parameters based on negative anti-VMX-C001 Abs screened animals

**: mean parameters based on negative anti-FactorX Abs screened animals

Supplementary Methods

Sample processing by parameter

Anti-drug antibodies

An exact volume of 0.9 mL of blood was transferred into a tube containing 100 µL of 3.2% sodium citrate (one part citrate, nine parts of blood). The tube was then gently inverted manually (up and down) at least five times. Tubes were kept upright without shaking and placed on wet ice pending centrifugation. The samples were centrifuged (3000*g* for 10 minutes at 4°C) within 2 hours of sample collection and the resultant plasma was separated, transferred to labelled propylene tubes and stored at −80°C within 90 minutes of centrifugation, and until analysis (Ardena Bioanalysis, Assen, Netherlands).

Toxicokinetics

An exact volume of 0.3 mL of blood was transferred into a tube containing 33.3 µL of 3.2% sodium citrate (one part citrate, nine parts of blood). The tube was then gently inverted manually (up and down) at least five times. Tubes were kept upright without shaking and placed on wet ice pending centrifugation within 90 minutes after blood collection. Blood was centrifuged at 3000*g* for 10 minutes under refrigerated conditions (set to maintain +4°C) within 90 minutes of sample collection. The plasma was kept refrigerated until transfer of the whole volume in a single aliquot containing at least 100 µL of plasma. Plasma aliquots were stored within 2 hours of centrifugation at −80°C (or placed on dry ice within 2 hours before storage at −80°C).

TAT and D-dimers

SCAT-1 tubes were placed at room temperature at least 30 minutes before blood collection. Venous blood was collected, according to the following procedure:

1. The first 0.5–1.2 mL of blood were collected with an empty syringe, and the syringe was removed from the needle taking care to keep the needle into the vein. This first syringe was either used for TK and /or ADA or discarded.
2. When applicable, blood was collected for thrombin generation
3. With the needle still in the vein and as soon as possible, an adaptor was connected for dedicated vacuum sampling.
4. Blood was collected for TAT and D-dimers in a vacuum SCAT-1 up to 0.6 mL.
5. The tube was then disconnected from the adaptor and mixed by gently inverting it at least five times.
6. Blood was collected for other investigations as applicable.

Samples were kept upright without shaking at room temperature until centrifugation. The samples were centrifuged at 1500*g* for 10 minutes at room temperature within 1 hour of sample collection. The resulting plasma was transferred into a new tube and centrifuged at 1500*g* for 10 minutes at room temperature. Plasma was divided into two aliquots, as follows:

1. Aliquot 1, at least 100 µL, for D-dimer measurement
2. Aliquot 2, at least 100 µL, for TAT complex measurement.

Plasma samples were frozen immediately in liquid nitrogen, kept on dry ice and stored within 45 minutes after the second centrifugation in a freezer set to maintain −80°C. The samples were shipped to Ardena (Assen, Netherlands) for analysis.

Thrombin generation

Samples were centrifuged (3000*g* for 10 minutes at room temperature [RT]) within 1 hour of sample collection, then transferred into a new tube and centrifuged at 10000*g* for 5 minutes at RT to separate plasma from platelets. The resultant plasma aliquots were transferred into individual tubes and stored at −80°C within 2 hours of the second centrifugation step, until dispatched for analysis.

Rivaroxaban

An exact volume of 0.3 mL was transferred into a tube containing 33.3 µL of 3.2% sodium citrate (1 part citrate, 9 parts blood). The tube was then gently inverted (up and down) at least five times. Tubes were kept upright without shaking and placed on wet ice pending centrifugation. The samples were centrifuged (3000*g* for 10 minutes under refrigerated conditions, set to maintain +4°C) within 90 minutes after collection and the resultant plasma was separated and transferred to uniquely labelled clear polypropylene tubes, and frozen within 2 hours after centrifugation over dry ice or in a freezer set to maintain −80°C.

Supplementary Figure S1. Thrombin generation in Sprague-Dawley rats (n=20 per dose) and cynomolgus monkeys (n=6 per dose) treated with VMX-C001. The bars show mean values, with individual values shown as open circles.

A) **B**)

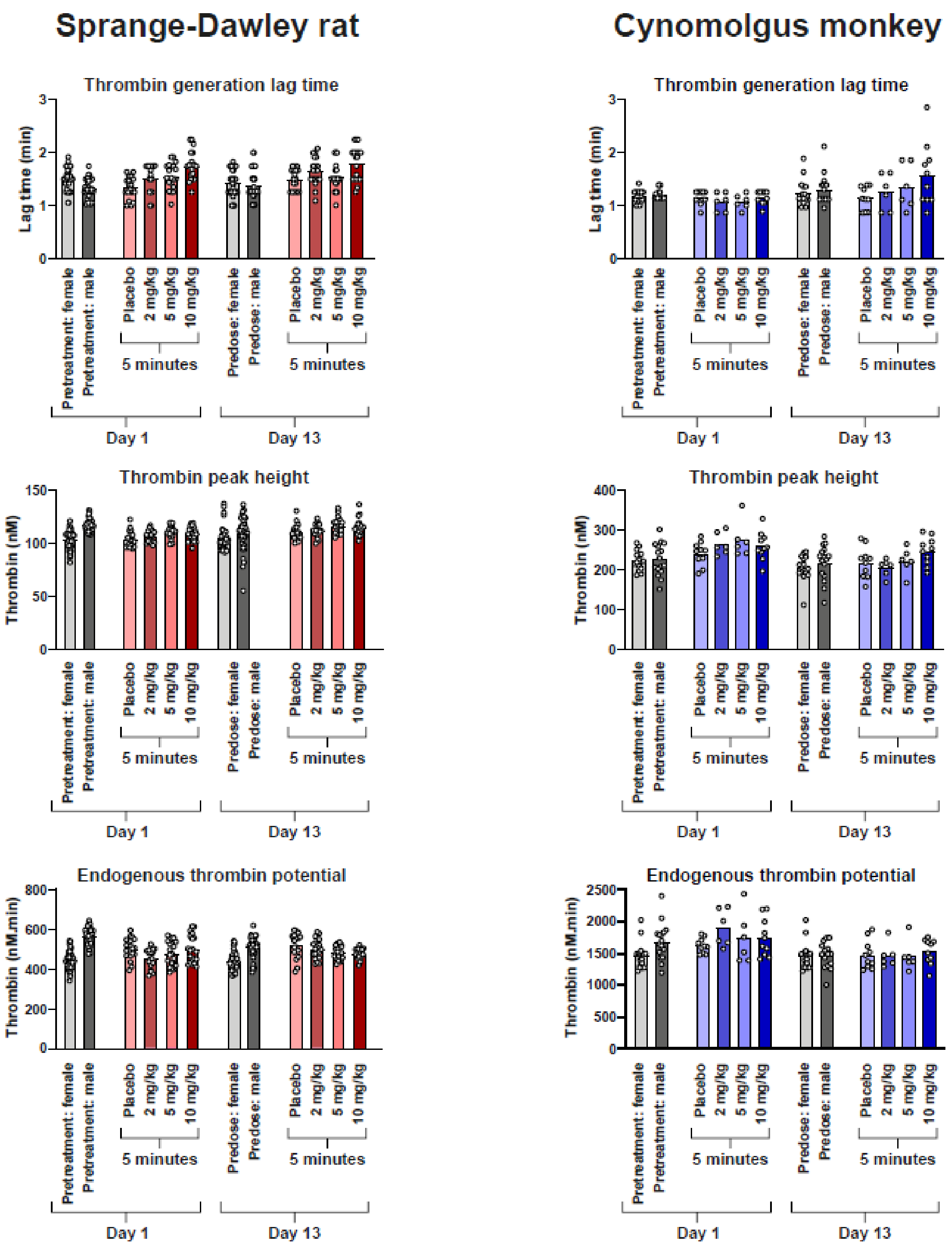

Data from female and male animals are shown together for all timepoints except for Pretreatment.

Supplementary Figure S2. D-dimer and TAT complexes upon bypassing of rivaroxaban with VMX-C001 in cynomolgus monkeys (n=5). The bars show mean values, with individual values shown as open circles.

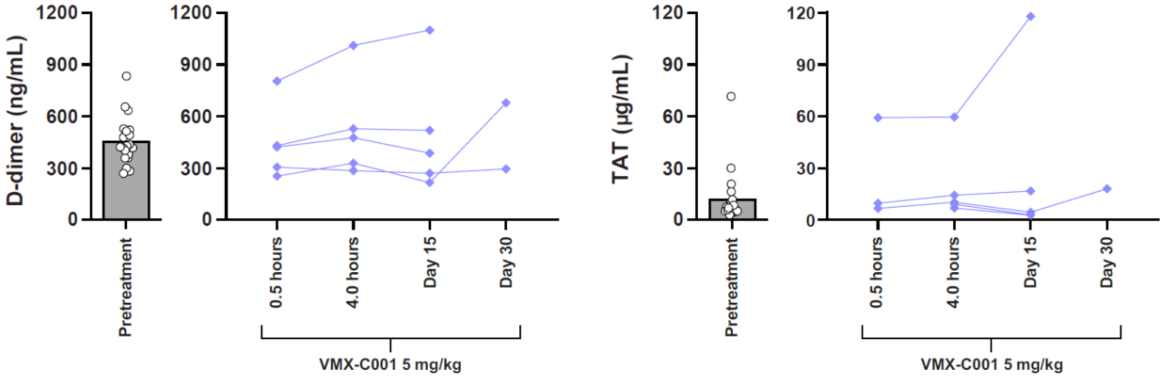

Supplement: Supplementary file 1 [file pharmaceutics-18-01210-s001.zip › pharmaceutics-4516896-supplementary.pdf]
